# Sensitive and Direct Detection of Receptor Binding Specificity of Highly Pathogenic Avian Influenza A Virus in Clinical Samples

**DOI:** 10.1371/journal.pone.0078125

**Published:** 2013-10-18

**Authors:** Tadanobu Takahashi, Tatsuya Kawakami, Takashi Mizuno, Akira Minami, Yuko Uchida, Takehiko Saito, Shigeyuki Matsui, Makoto Ogata, Taichi Usui, Nongluk Sriwilaijaroen, Hiroaki Hiramatsu, Yasuo Suzuki, Takashi Suzuki

**Affiliations:** 1 Department of Biochemistry, School of Pharmaceutical Sciences, University of Shizuoka, Shizuoka, Shizuoka, Japan; 2 Viral Disease and Epidemiology Research Division, National Institute of Animal Health, National Agriculture and Food Research Organization (NARO), Tsukuba, Ibaraki, Japan; 3 Zoonotic Diseases Collaboration Center (ZDCC), Bangkok, Thailand; 4 Shizuoka Prefectural Livestock Institute, Swine and Poultry Research Center, Kikugawa, Shizuoka, Japan; 5 Department of Chemistry and Biochemistry, Fukushima National College of Technology, Iwaki, Fukushima, Japan; 6 Department of Bioscience, Graduate School of Science and Technology, Shizuoka University, Shizuoka, Shizuoka, Japan; 7 Department of Applied Biological Chemistry, Faculty of Agriculture, Shizuoka University, Shizuoka, Shizuoka, Japan; 8 Health Science Hills, College of Life and Health Sciences, Chubu University, Kasugai, Aichi, Japan; 9 Faculty of Medicine, Thammasat University, Pathumthani, Thailand; Centers for Disease Control and Prevention, United States of America

## Abstract

Influenza A virus (IAV) recognizes two types of *N*-acetylneuraminic acid (Neu5Ac) by galactose (Gal) linkages, Neu5Acα2,3Gal and Neu5Acα2,6Gal. Avian IAV preferentially binds to Neu5Acα2,3Gal linkage, while human IAV preferentially binds to Neu5Acα2,6Gal linkage, as a virus receptor. Shift in receptor binding specificity of avian IAV from Neu5Acα2,3Gal linkage to Neu5Acα2,6Gal linkage is generally believed to be a critical factor for its transmission ability among humans. Surveillance of this shift of highly pathogenic H5N1 avian IAV (HPAI) is thought to be a very important for prediction and prevention of a catastrophic pandemic of HPAI among humans. In this study, we demonstrated that receptor binding specificity of IAV bound to sialo-glycoconjugates was sensitively detected by quantifying the HA gene with real-time reverse-transcription-PCR. The new assay enabled direct detection of receptor binding specificity of HPAIs in chicken clinical samples including trachea and cloaca swabs in only less than 4 h.

## Introduction

 Influenza A virus (IAV) initiates infection through one of sialic acids, *N*-acetylneuraminic acid (Neu5Ac), bonded to terminal galactose (Gal) of glycoconjugates on the host cell surface. A membrane glycoprotein of IAV, hemagglutinin (HA), recognizes two types of linkages between Neu5Ac and Gal, Neu5Acα2,3Gal and Neu5Acα2,6Gal. IAV has a wide spectrum of hosts including humans, birds, pigs and horses. Human IAV shows preferential binding to Neu5Acα2,6Gal, which is abundantly expressed in respiratory tracts of humans. On the other hand, avian IAV shows preferential binding to Neu5Acα2,3Gal, which is abundantly expressed in respiratory and intestinal tracts of birds [1-3]. These receptor binding specificities of IAV by sialic acid linkage are thought to be an important determinant in host specificity. Furthermore, shift in receptor binding specificity of avian IAV from Neu5Acα2,3Gal to Neu5Acα2,6Gal has been reported to be one of major factors for acquisition of avian IAV transmission ability among humans [4,5]. Infection with the highly pathogenic H5N1 avian IAV (HPAI) has a high fatality rate (approximately 60% as of 2012) even in cases of human infection [6]. Shift in receptor binding specificity of HPAI is thought to increase transmission potential among humans, which may lead to a catastrophic pandemic. Surveillance of the shift in receptor binding specificity of HPAI is thought to be very important for prediction and prevention of an HPAI pandemic among humans.

 Receptor binding specificity of influenza viruses is mainly determined by a hemagglutination assay with resialylated erythrocytes [7,8], a solid-phase virus binding assay with sialo-glycoconjugates [9-11], a thin layer chromatography (TLC) virus overlay assay with sialo-glycoconjugates [11-14], and by glycoarray analysis with synthetic and natural glycans [15-17]. Bound viruses to sialo-glycoconjugates are detected by using specific anti-influenza virus antibodies and an enzyme-labeled secondary antibody [10,12,15,16] or labeling with fluorescent dyes such as Alexa 488 [17]. Additionally, high titer of concentrated IAV, which is propagated in embryonated eggs or cultured cells from seed virus, is required to determine receptor binding specificity in these assays. This process takes several days or more than one week. If there is no appropriate antibody against the IAV strain of interest, we must prepare a specific antibody. Our previous studies have shown that glycopolymers bind strongly with IAV, possibly because of the cluster effect of sialo-glycoconjugates. Glycopolymers are ϒ-polyglutamic acid polymers containing *N*-acetyllactosamine (LacNAc) glycoconjugates possessing Neu5Acα2,3Gal or Neu5Acα2,6Gal [18,19]. However, even when glycopolymers are utilized, a high titer of IAV is required for determination of the receptor binding specificity of IAV. Since the traditional assay requires a long time to complete detection for receptor binding specificity of IAV, it is not easily applied for screening of the shift in receptor binding specificities of many HPAI stains.

 In this study, we tried to directly detect receptor binding specificity of avian IAV in clinical samples by improving the detection sensitivity of IAV bound to glycopolymers. Direct detection from clinical samples should not require a virus replication process, leading to much reduction of the time to complete detection of receptor binding specificity. To improve detection intensity of IAV bound to glycopolymers immobilized on a microplate, we quantified the HA gene of IAV using real-time PCR and one-step reverse-transcription (RT)-PCR. The new assay enabled detection of receptor biding specificities of IAVs with a minimum 50% egg infectious dose (EID_50_) of 10^4.02^. The new assay also enabled direct detection of receptor binding specificity of HPAIs in clinical samples such as trachea swab and cloaca swab samples, without influence of the swab itself. Although the traditional assay usually required more than one week, the new assay finished the detection in only less than 4 h directly from clinical samples.

## Materials and Methods

### Viruses, glycopolymers, and swab samples

 IAVs A/Memphis/1/1971 (H3N2) (M71) and A/duck/Hong Kong/313/4/1978 (H5N3) (D313) were propagated in 10-day-old embryonated hen’s eggs for 2 days at 34 °C and were concentrated by centrifugation. 

ϒ-Polyglutamic acid polymers containing 5-aminopentyl β-LacNAc (PGA), 5-aminopentyl β-Neu5Acα2,3LacNAc (α2,3PGA), or 5-aminopentyl β-Neu5Acα2,6LacNAc (α2,6PGA) were synthesized as described previously [18]. The degree of polymerization (DP) of glutamic acid residues is 6557. Molecular weights of PGA, α2,3PGA, and α2,6PGA were 2,200,000, 3,000,000 and 3,000,000, respectively. The degree of substitution of sialo-sugar derivatives based on DP of non-sugar ϒ-PGA is 43% in both α2,3PGA and α2,6PGA (Figure S1). 

Swabs were obtained from five white leghorn chickens (brand, Julia) bred in Shizuoka Prefectural Livestock Institute, Swine and Poultry Research Center. Each poultry trachea swab sample was a cotton-tipped swab suspended in 0.5 ml phosphate-buffered saline (PBS; pH 7.2, 131 mM NaCl, 14 mM Na_2_HPO_4_, 1.5 mM KH_2_PO_4_, and 2.7 mM KCl). Each poultry cloaca swab sample was a cotton-tipped swab suspended in 1.5 ml phosphate-buffered saline. Trachea swabs and cloaca swabs from HPAI-infected chickens were given from swab samples reported previously [20]. HPAI strains, A/chicken/Shimane/1/2010 (H5N1) (CS10) and A/chicken/Miyazaki/S4/2011 (H5N1) (CM11), were used. Accession numbers in GenBank are AB684240 for CS10 HA and AB684242 for CM11 HA. Both HA genes are included in clade 2. 3. 2. 1. of HPAI H5HA genes. HA sequences of the two viruses are closely related (only two differences at the amino acid level). The EID_50_ per 200 μl in the trachea swab and the cloaca swab containing CS10 were 10^6.53^ and 10^4.38^, respectively. The EID_50_ per 200 μl in the trachea swab and the cloaca swab containing CM11 were 10^5.46^ and 10^4.32^, respectively. We used these four HPAI-positive samples. All animal experiments were carried out in biosafety level 3 facilities at the National Institute of Animal Health, Japan and were approved by the Ethics Committee of the institute.

### Hemagglutination assay

 Fifty microliters per well of 2-fold serially diluted virus suspensions were mixed with 50 μl of 0.5% (v/v) guinea pig red blood cells on a 96-well U-bottom microplate. After incubation at 4 °C for 2 h, hemagglutination unit (HAU) was measured.

### Detection of Neu5Acα2,3Gal or Neu5Acα2,6Gal glycoconjugates on PGA by lectins

 Fifty nanograms per well of α2,3PGA, α2,6PGA or PGA was incubated on a Corning 96-well clear flat-bottom polystyrene Universal-BIND microplate (Corning Life Sciences, Lowell, MA, USA) at 4 °C for 2 h and immobilized by irradiation of UV at 256 nm for 1 min. After washing with PBS 5 times, the wells were blocked by incubation of 300 μl/well of 1% lipid-free bovine serum albumin (BSA) in PBS at 4 °C overnight. After washing with PBS 5 times, the wells were incubated with 100 μl/well (5 μg/ml) of biotinylated *Maackia amurensis* lectin (MAM; J-OIL MILLS, Tokyo, Japan) or biotinylated *Sambucus sieboldiana* lectin (SNA; J-OIL MILLS, Tokyo, Japan) at room temperature for 2 h. After washing with PBS 5 times, the wells were incubated with 130 μl/well of horseradish peroxidase (HRP)-labeled streptavidin (SIGMA-ALDRICH, St. Louis, MO, USA). Finally, the wells were colored as described previously [11,18].

### Measurement of receptor binding specificity of IAVs by real-time one-step RT-PCR

 Two hundred nanograms per well of α2,3PGA or α2,6PGA was incubated on a Corning 96-well clear flat-bottom Polystyrene Universal-BIND microplate at 4°C for 2 h and immobilized by irradiation of UV at 256 nm for 1 min. After washing with PBS 5 times, the wells were blocked by incubation of 300 μl/well of 1% lipid-free BSA in PBS at 4 °C overnight. After washing with PBS containing 0.01% Tween20 10 times, 50 μl/well of D313 or M71 (2° HAU) in PBS containing 0.1% lipid-free BSA was incubated 4 °C for 2 h. A QIAGEN RNeasy Mini kit (QIAGEN, Venlo, Netherlands) was used to extract viral RNA of IAVs bound to α2,3PGA or α2,6PGA. After washing with PBS 10 times, the wells were mixed with 200 μl/well of RLT buffer of the QIAGEN RNeasy Mini kit. Then 150 μl of RLT buffer and 100 μl of sterilized distilled water were added to the buffer. After mixing vigorously, 250 μl of 99.5% ethanol was added. The mixture was poured into the cartridge of the QIAGEN RNeasy Mini kit. After centrifugation at 8,000 ×*g* for 1 min, 700 μl of RW1 buffer was poured into the cartridge. After centrifugation at 8,000 ×*g* for 1 min, 500 μl of RPE buffer was poured into the cartridge. After centrifugation at 8,000 ×*g* for 1 min, 500 μl of RPE buffer was poured into the cartridge again. Viral RNA in the cartridge was eluted with 30 μl of RNase-free water. The RNA was stored at -80 °C until use. Viral HA cDNA copies were measured by using real-time PCR equipment (Light Cycler ver.2.0; Roche, Hague Road, IN, USA), a One Step SYBR PrimeScript RT-PCR PLUS RT-PCR kit (Perfect Real Time) (TaKaRa Bio, Shiga, Japan), and primer pairs, 5’-ATGGCAGGGAATGGTAGA-3’ and 5’-TTGCCTTTTGAGTGGATTC-3’ for the D313 HA gene and 5’-CCTCATCGAATCCTTGATG-3’ and 5’-GGGTAACAGTTGCTGAAAGC-3’ for the M71 HA gene. Primer pairs for the D313 HA gene amplify 95 base pairs between 1093 and 1187 at D313 HA nucleotide positions, which are located at the 5’ region side of the HA2 gene. These primers were designed on the basis of conserved nucleotide sequences among the D313 HA gene (accession number AB542809) in 1978, duck H5N3 HA gene (EF597247) in 1976, and HPAI H5N1 HA genes from various hosts including chickens (DQ076201, DQ083551, and DQ083565), white peafowls (DQ083573), mynas (DQ083585), pigeons (DQ083583), crows (DQ083563), ducks (DQ083581), geese (AY651332), tigers (AY646167, AY842935, AY972540, AY972541, AY866475, and AY972539), leopards (AY646175), and humans (AY627885, AY626143, AY577314, and AY555153) in 1976 and 2004. Primer pairs for the M71 HA gene amplify 134 base pairs between 240 and 373 at M71 HA nucleotide positions, which are located at the 5’ region side of the HA1 gene. These primers are also used as sequencing primers for the human IAV H3N2 HA gene. Two microliters of the viral RNA samples was added to the RT-PCR solution (20 μl/capillary) as a RT-PCR template. For RT reaction, the solution in the capillary was incubated at 42 °C for 5 min (20 °C/sec) and heated at 95 °C for 10 sec (20 °C/sec). For PCR reaction (60 cycles), after denaturing at 95 °C for 5 sec (20 °C/sec), the solution in the capillary was heated at 95 °C for 5 sec (20 °C/sec), annealed at 55 °C for 15 sec (20 °C/sec), and extended at 66 °C for 15 sec (20 °C/sec). Standard curve of HA cDNA copies (cycle values v.s. HA cDNA copies) was made by use of the expression vector pCAGGS containing the D313 HA gene between the *Sma* I site and *Xho* I site or the M71 HA gene between the *Kpn* I site and *Sma* I site [21] as a template of real-time PCR.

 To investigate influence of contents in poultry swabs on this receptor binding specificity assay, 25 μl/well of D313 or M71 (2° HAU) in PBS containing 0.1% lipid-free BSA was mixed with 25 μl/well of a 5-dilution suspension of the supernatant of the trachea swab or cloaca swab after spin-down for 5 min. Fifty microliters per well of the mixture was incubated on a microplate that wells were immobilized with 200 ng/well of α2,3PGA or α2,6PGA and then blocked with 1% lipid-free BSA (300 μl/well). For the receptor binding specificity assay of HPAIs in poultry swabs, supernatants of trachea swabs or cloaca swabs after spin-down for 5 min were two-times suspended in PBS. On a microplate that was immobilized with 200 ng/well of α2,3PGA or α2,6PGA and then blocked with 1% lipid-free BSA (300 μl/well), the wells were incubated with 50 μl/well of the swab supernatants at room temperature for 2 h. The experiment using HPAIs was performed in a biosafety level 3 facility until addition of RLT buffer to the wells. The viral RNA of viruses bound to α2,3PGA or α2,6PGA were extracted as described above. The amount of HPAI HA genes was measured by real-time one-step RT-PCR using the primer pair 5’-ATGGCAGGGAATGGTAGA-3’ and 5’- TCTATTGCCTTTTGAGTAGATTC-3’. The sequence of the former primer is the same as that of the forward primer for the D313 HA gene. The latter primer was designed on approximately the nucleotide location of the reverse primer for the D313 HA gene (addition of 4 nucleotides and replacement of 1 nucleotide), because a base “C” at nucleotide position 1174 in D313 HA (D313 HA numbering) was substituted to a base “T” in both HPAI HAs. Primer pairs for the HPAI HA gene amplify 108 base pairs (plus 9 nucleotides in the trypsin-sensitive cleavage site) between nucleotide positions 1093 and 1191 (D313 HA numbering). The entire gene of HPAI HA cDNA was amplified by PCR (20 sec at 94 °C for denaturing, 30 sec at 58 °C for annealing, and 7 min at 72 °C for extension) using DyNAzyme EXT DNA polymerase (Thermo Fisher Scientific K.K., Yokohama, Japan) and primer pair 5’-TATTCGTCTCAGGGAGCAAAAGCAGGGG-3’ and 5’- ATATCGTCTCGTATTAGTAGAAACAAGGGTGTTTT-3’. The amplified HA genes were purified from band cut at approximately 1.8 k base pairs on 1% agarose gel, and the amount of HA genes was quantified by using Nanodrop 1000 (Thermo Fisher Scientific K.K., Yokohama, Japan). Standard curve of HPAI HA cDNA copies (cycle values v.s. HA cDNA copies) was made by use of the amplified HA genes as a template of real-time PCR.

### Solid-phase virus binding assay by using anti-IAV antibody

A microplate with α2,3PGA (200 ng/well) was blocked with 1% lipid-free BSA in PBS at 4 °C overnight and was then incubated with different titers of avian IAV D313 (two serial dilutions from 2^4^ to 2^-3^ HAU/50 μl) for 2 h at 4 °C. Viruses bound to α2,3PGA were detected by rabbit anti-D313 polyclonal antibody and HRP-labeled goat anti-rabbit IgG antibody. Finally, amounts of viruses bound to α2,3PGA were measured by development of *O*-phenylenediamine (OPD), as previously reported [11,18].

## Results

### Binding specificity of Neu5Acα2,3Gal and Neu5Acα2,6Gal on a PGAs-immobilized microplate by lectins

**Figure 1:**
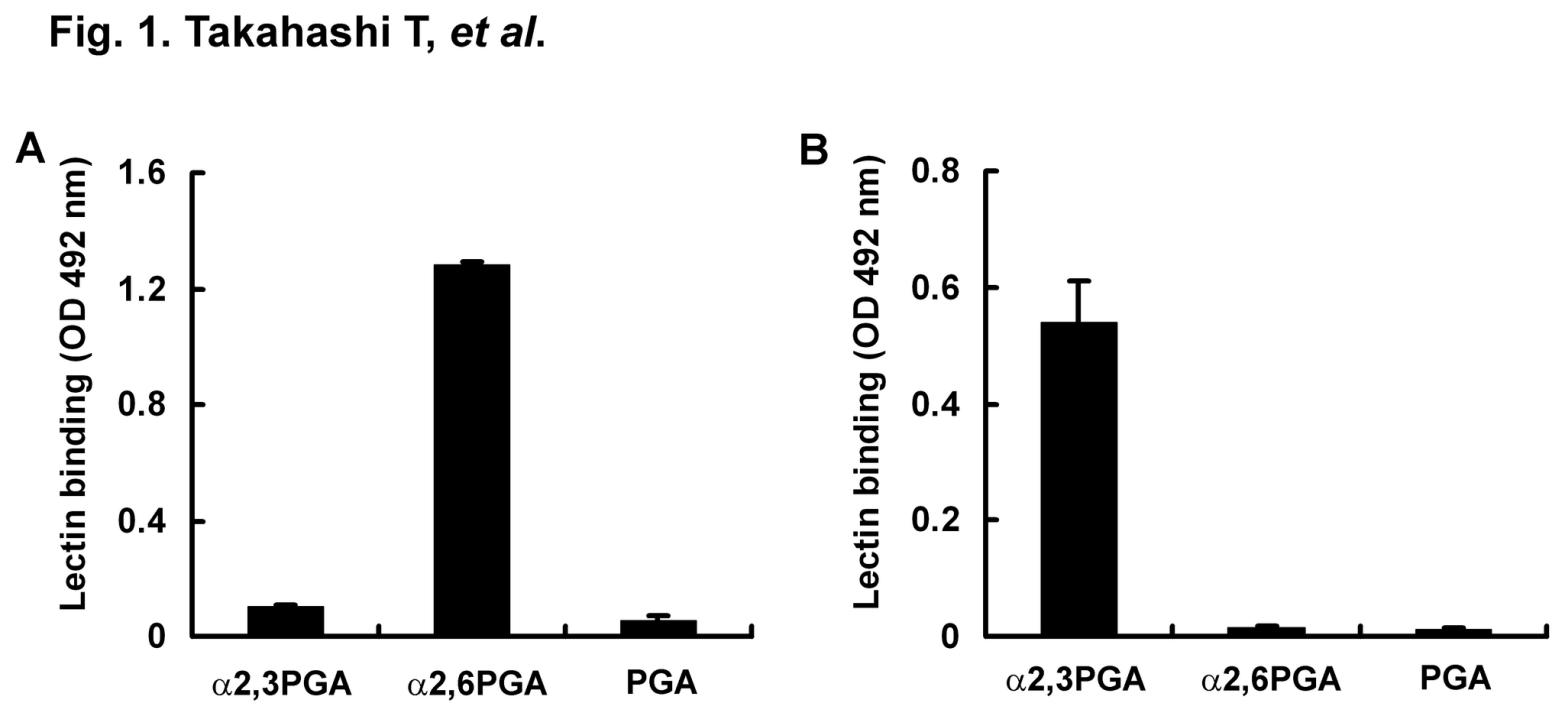
Lectin binding to PGAs immobilized on a microplate. α2,3PGA, α2,6PGA, and PGA containing an LacNAc chain (each 50 ng/well) on a microplate were reacted with biotinylated lectins, SNA (A) and MAM (B). Each value (standard error; S.E.) is the average from three independent experiments. OD, optical density.

 α2,3PGA (possessing Neu5Acα2,3LacNAc), α2,6PGA (possessing Neu5Acα2,6LacNAc), and PGA (possessing LacNAc) were covalently immobilized on a microplate by irradiation of UV (Figure S1). We checked whether lectins could recognize terminal sialic acid linkages of glycoconjugates on the immobilized PGAs, Neu5Acα2,3Gal and Neu5Acα2,6Gal. After blocking of lipid-free BSA, the wells were incubated with biotinylated SNA or biotinylated MAM. Lectins that bound to glycoconjugates of PGAs were detected with HRP-labeled streptavidin. SNA, which could recognize Neu5Acα2,6Gal, bound to α2,6PGA only (Figure 1A). On the other hand, MAM, which could recognize Neu5Acα2,3Gal, bound to α2,3PGA only (Figure 1B). This result indicates that glycoconjugates of the immobilized PGAs maintain sialic acid linkages that lectins can recognize and that the immobilized PGAs are useful for detection of IAV receptor binding specificity.

### Highly sensitive detection of receptor binding specificity of IAVs by using real-time PCR

**Figure 2:**
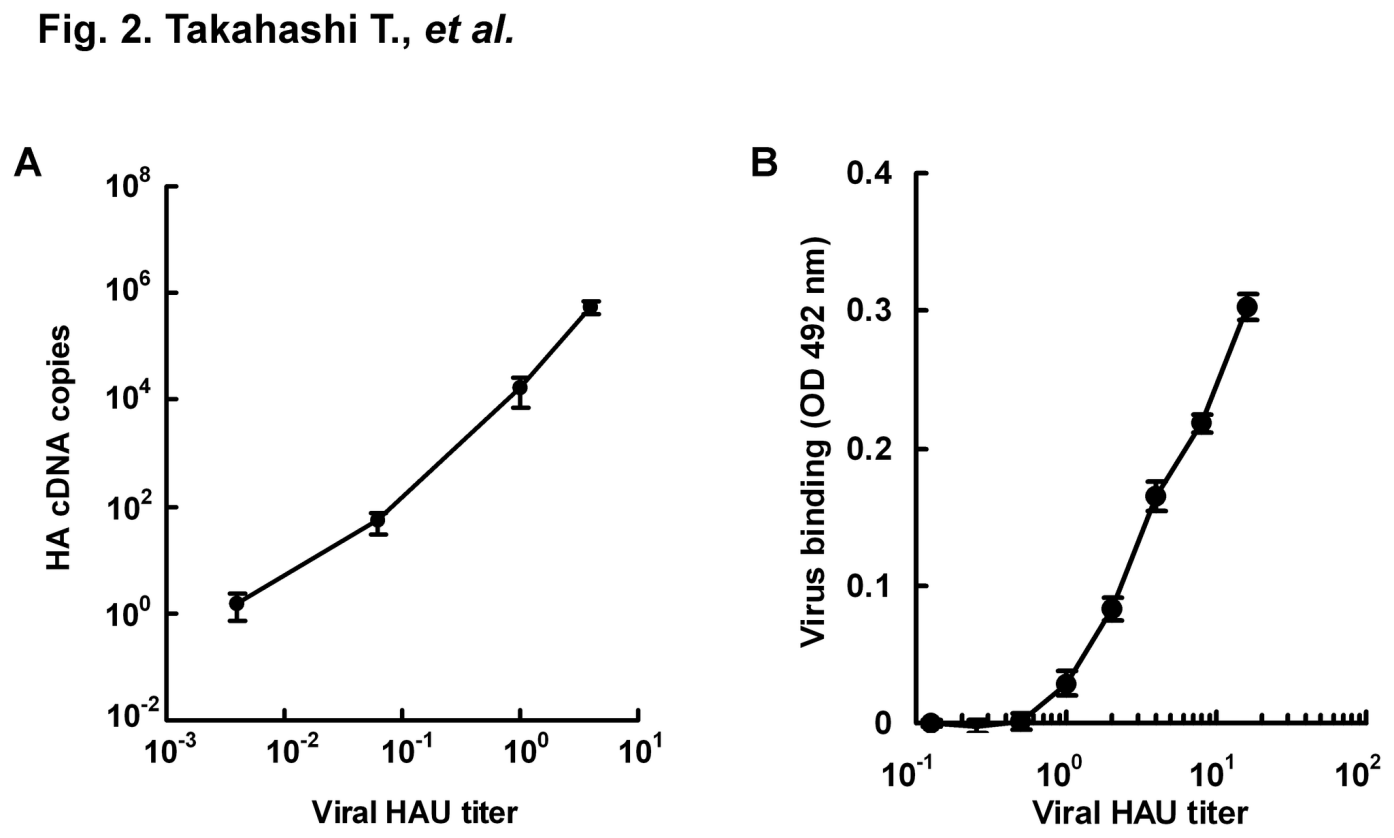
Detection of virus binding to α2,3PGA by real-time PCR and by solid-phase virus binding assay. A, A microplate was immobilized with 200 ng/well of α2,3PGA and then blocked with 1% lipid-free BSA in PBS at 4 °C overnight. Different titers of avian IAV D313 (2^2^, 2°, 2^-4^, and 2^-8^ HAU/50 μl) were incubated for 2 h at 4 °C on microplates that were immobilized with α2,3PGA. Viral RNAs of viruses bound to α2,3PGA were extracted. Amount of HA cDNA copies was measured by using real-time PCR. Each value (S.E.) is the average from three independent experiments. Receptor binding of D313 to α2,3PGA was able to be linearly measured within the range of 2^2^ to 2^-4^ HAU from a correlation factor of more than 0.99. B, A microplate was immobilized with 200 ng/well of α2,3PGA and then blocked with 1% lipid-free BSA in PBS at 4 °C overnight. Different titers of avian IAV D313 (2^4^ to 2^-3^ HAU/50 μl) were incubated on a microplate. Viruses bound to α2,3PGA were detected by rabbit anti-D313 polyclonal antibody.

**Figure 3:**
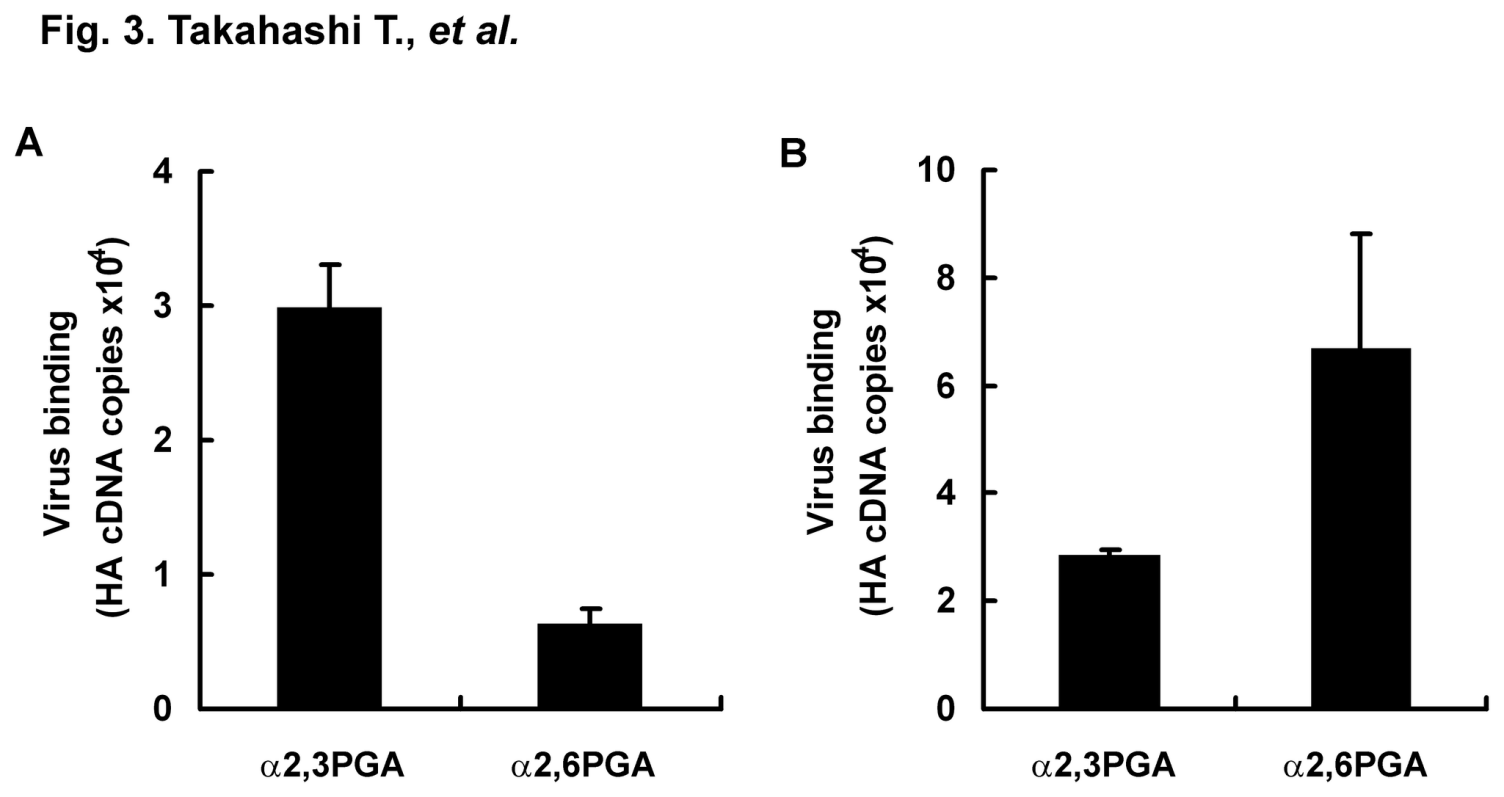
Detection of receptor binding specificity of avian and human viruses by real-time PCR. A microplate was immobilized with 200 ng/well of α2,3PGA or α2,6PGA and then blocked with 1% lipid-free BSA in PBS at 4 °C overnight. Avian IAV D313 (A) and human IAV M71 (B) (2° HAU, 50 μl/well) were incubated for 2 h at 4 °C on microplates that were immobilized with α2,3PGA or α2,6PGA. Viral RNAs of viruses bound to α2,3PGA or α2,6PGA were extracted. Viruses that bound to α2,3PGA or α2,6PGA were measured as copy number of HA cDNA by using real-time PCR. Each value (S.E.) is the average from three independent experiments. The general property for receptor binding specificities (preferential binding of avian IAV to Neu5Acα2,3Gal and of human IAV to Neu5Acα2,6Gal) was detected.

 To improve detection sensitivity of receptor binding specificity of IAVs, we tried to measure genes of virus bound to glycoconjugates possessing Neu5Acα2,3Gal and Neu5Acα2,6Gal by using real-time PCR. Wells with immobilized α2,3PGA were blocked with lipid-free BSA and incubated with 2^2^, 2°, 2^-4^, or 2^-8^ HAU/50 μl of avian IAV strain D313, which preferentially recognized Neu5Acα2,3Gal in a solid-phase virus binding assay [18]. Materials other than the viruses that had bound to α2,3PGA were washed out 5 times before extraction of viral RNA. HA cDNA copies of D313 bound to α2,3PGA were measured by one-step RT-PCR. Correlation factors between HAU and HA cDNA copies were 0.997 and 0.982 within the ranges of 2^2^ to 2^-4^ HAU and 2^2^ to 2^-8^ HAU, respectively. We estimated that receptor binding of D313 to α2,3PGA was able to be linearly measured within the range of 2^2^ to 2^-4^ HAU from a correlation factor of more than 0.99 (Figure 2A). Here, we tried highly sensitive detection of receptor binding specificity of IAVs at 2° HAU. Usually, when a solid-phase virus binding assay and a TLC virus binding assay are performed using this titer, receptor binding specificities between Neu5Acα2,3Gal and Neu5Acα2,6Gal cannot be detected because of the weak signal of virus binding to glycoconjugates. Wells with immobilized α2,3PGA or α2,6PGA were blocked with lipid-free BSA and incubated with 2° HAU/50 μl of D313 or human IAV strain M71, which preferentially recognized Neu5Acα2,6Gal in a solid-phase virus binding assay [18]. HA cDNA copies of bound D313 and M71 were measured by using real-time PCR. Melting temperatures of all PCR products were confirmed to be similar to approximately 81 °C in a positive control (standard curve) of D313 and M71, indicating that PCR products detected were the same HA gene. Average HA cDNA copies of D313 were 2.99 ×10^4^ (S.E. ± 3.15 ×10^3^) for α2,3PGA and 6.37 ×10^3^ (S.E. ± 1.09 ×10^3^) for α2,6PGA. HA cDNA copies of M71 were 2.86 ×10^4^ (S.E. ± 9.56 ×10^2^) for α2,3PGA and 6.68 ×10^4^ (S.E. ± 2.13 ×10^4^) for α2,6PGA. D313 preferentially bound to α2,3PGA (Figure 3A), while M71 preferentially bound to α2,6PGA (Figure 3B), indicating that this assay enables detection of receptor binding specificity of avian and human IAVs. For detection of receptor binding specificity of IAV, a traditional solid-phase virus binding assay using α2,3PGA and α2,6PGA has usually been performed with 2^5^ to 2^8^ HAU of IAV [18,19,22]. Virus titer in the new assay described in this paper showed 32- to 256-times higher sensitivity than the conventional virus titer in a traditional assay.

 We tried a solid-phase virus binding assay with α2,3PGA. A microplate with α2,3PGA was incubated with two serial dilutions from 2^4^ to 2^-3^ HAU/50 μl of D313. D313 bound to α2,3PGA was detected by rabbit anti-D313 antibody. IAV binding signal was not detected under the 2^1^ HAU because of less than 0.1 at absorbance (Figure 2B). The result indicates that the new assay is sensitive compared to traditional assays, at least solid-phase virus binding assay.

### Direct detection of receptor binding specificity of avian IAVs from clinical samples

**Figure 4:**
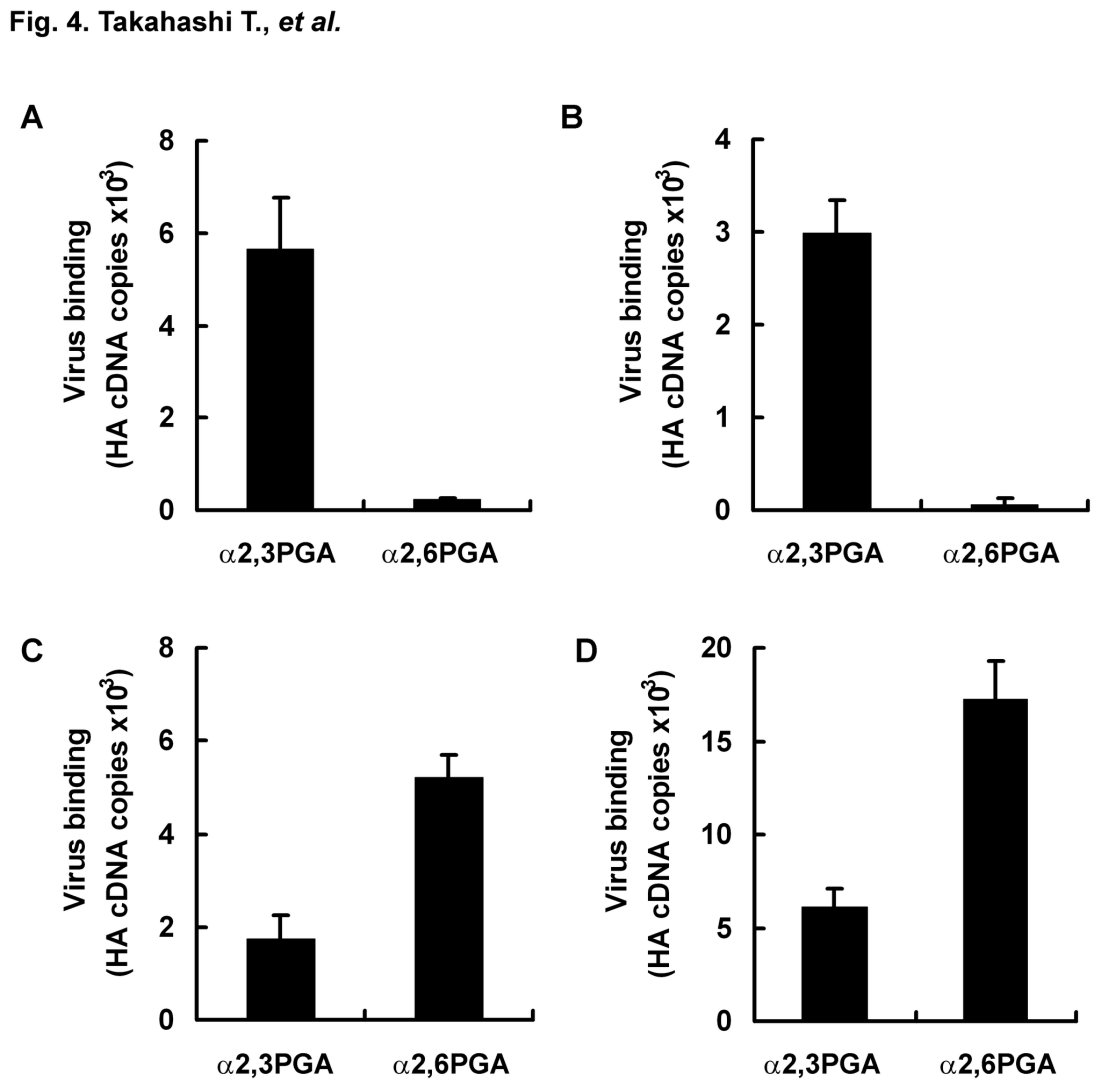
Influence of swabs from chickens on the real-time PCR assay for receptor binding specificity. A microplate was immobilized with 200 ng/well of α2,3PGA or α2,6PGA and then blocked with 1% lipid-free BSA in PBS at 4 °C overnight. Avian IAV D313 (A and B) or human IAV M71 (C and D) (2° HAU, 25 μl/well) was mixed with a 5-dilution suspension of a trachea swab (A and C) or cloaca swab (B and D) (25 μl/well) from healthy chickens. The mixtures (50 μl/well) were incubated for 2 h at 4 °C on microplates that were immobilized with α2,3PGA or α2,6PGA. Viral RNAs of viruses bound to α2,3PGA or α2,6PGA were extracted. Viruses that bound to α2,3PGA or α2,6PGA were measured as copy number of HA cDNA by using real-time PCR. The data (S.E.) is the average from three independent experiments. The trachea swab and cloaca swab had no influence on this assay.

 We tried to directly detect receptor binding specificity of avian IAV in poultry clinical samples including trachea swab and cloaca swab samples. To check influence of contents in poultry swabs on detection for receptor binding specificity of avian and human IAV, D313 and M71 were mixed with poultry trachea swabs or cloaca swabs obtained from five chickens. The supernatant after spin-down was used as a virus-swab suspension. Wells with immobilized α2,3PGA or α2,6PGA were blocked with lipid-free BSA and incubated with a virus-swab suspension (2^-1^ HAU/50 μl) of D313 or M71. HA cDNA copies of bound D313 and M71 were measured by using real-time PCR. Melting temperatures of all PCR products were confirmed to be similar to approximately 81°C in a positive control (standard curve) of D313 and M71. HA cDNA copies of D313 were 5.66 ×10^3^ (S.E. ± 1.12 ×10^3^) for α2,3PGA and 2.23 ×10^2^ (S.E. ± 2.75 ×10^1^) for α2,6PGA in the presence of a trachea swab, and they were 2.98 ×10^3^ (S.E. ± 3.56 ×10^2^) for α2,3PGA and 6.04 ×10^1^ (S.E. ± 6.55 ×10^1^) for α2,6PGA in the presence of a cloaca swab. HA cDNA copies of M71 were 1.73 ×10^3^ (S.E. ± 5.35 ×10^2^) for α2,3PGA and 5.22 ×10^2^ (S.E. ± 4.81 ×10^2^) for α2,6PGA in the presence of a trachea swab, and they were 6.10 ×10^3^ (S.E. ± 9.64 ×10^2^) for α2,3PGA and 1.72 ×10^4^ (S.E. ± 2.06 ×10^3^) for α2,6PGA in the presence of a cloaca swab. D313 preferentially bound to α2,3PGA in the presence of a trachea swab (Figure 4A) and a cloaca swab (Figure 4B), while M71 preferentially bound to α2,6PGA in the presence of a trachea swab (Figure 4C) and a cloaca swab (Figure 4D). These results, which are similar to the results of receptor binding specificity shown in Figure 3 in the absence of poultry swabs, indicate that poultry trachea and cloaca swabs have no effect on receptor binding specificity of avian and human IAVs for this assay. As negative controls, we assayed 10-diluted suspensions of three trachea and cloaca swabs without viruses on a microplate immobilized with α2,3PGA and α2,6PGA by RT-PCR. RT-PCR using all primer pairs tested showed no nonspecific detection for healthy chicken swabs (data not shown). Again, we performed additional run of experiments of D313 and M71 in the presence of a trachea swab or cloaca swab. These results indicated receptor binding specificities shown in Figure 4 (Table 1). They support the reliability of receptor binding specificities detected by the new assay.

**Table 1:** Reproduction of receptor binding specificities of D313 and M71 measured by the new assay in the presence of swabs.

Assay		HA cDNA copies ± S.E.			α2,3PGA / α2,6PGA		
runs		α2,3PGA		α2,6PGA		ratio	
D313 in the presence of trachea swab							
1st run	5.66 ×10^3^ ± 1.12 ×10^3^		2.23 ×10^2^ ± 2.75 ×10^1^				25.4
2nd run	2.86 ×10^4^ ± 8.96 ×10^3^		6.35 ×10^2^ ± 2.46 ×10^2^				45.0
D313 in the presence of cloaca swab							
1st run	2.98 ×10^3^ ± 0.36 ×10^2^		6.04 ×10^1^ ± 6.56 ×10^1^				49.3
2nd run	1.65 ×10^4^ ± 4.66 ×10^3^		3.83 ×10^2^ ± 7.29 ×10^1^				43.1
M71 in the presence of trachea swab							
1st run	1.73 ×10^3^ ± 5.35 ×10^2^		5.22 ×10^3^ ± 4.81 ×10^2^				0.33
2nd run	1.75 ×10^4^ ± 1.85 ×10^3^		3.37 ×10^4^ ± 3.31 ×10^3^				0.52
M71 in the presence of cloaca swab							
1st run	6.10 ×10^3^ ± 9.64 ×10^2^		1.72 ×10^4^ ± 2.06 ×10^3^				0.35
2nd run	1.67 ×10^4^ ± 4.83 ×10^3^		3.60 ×10^4^ ± 1.42 ×10^4^				0.46

**Figure 5:**
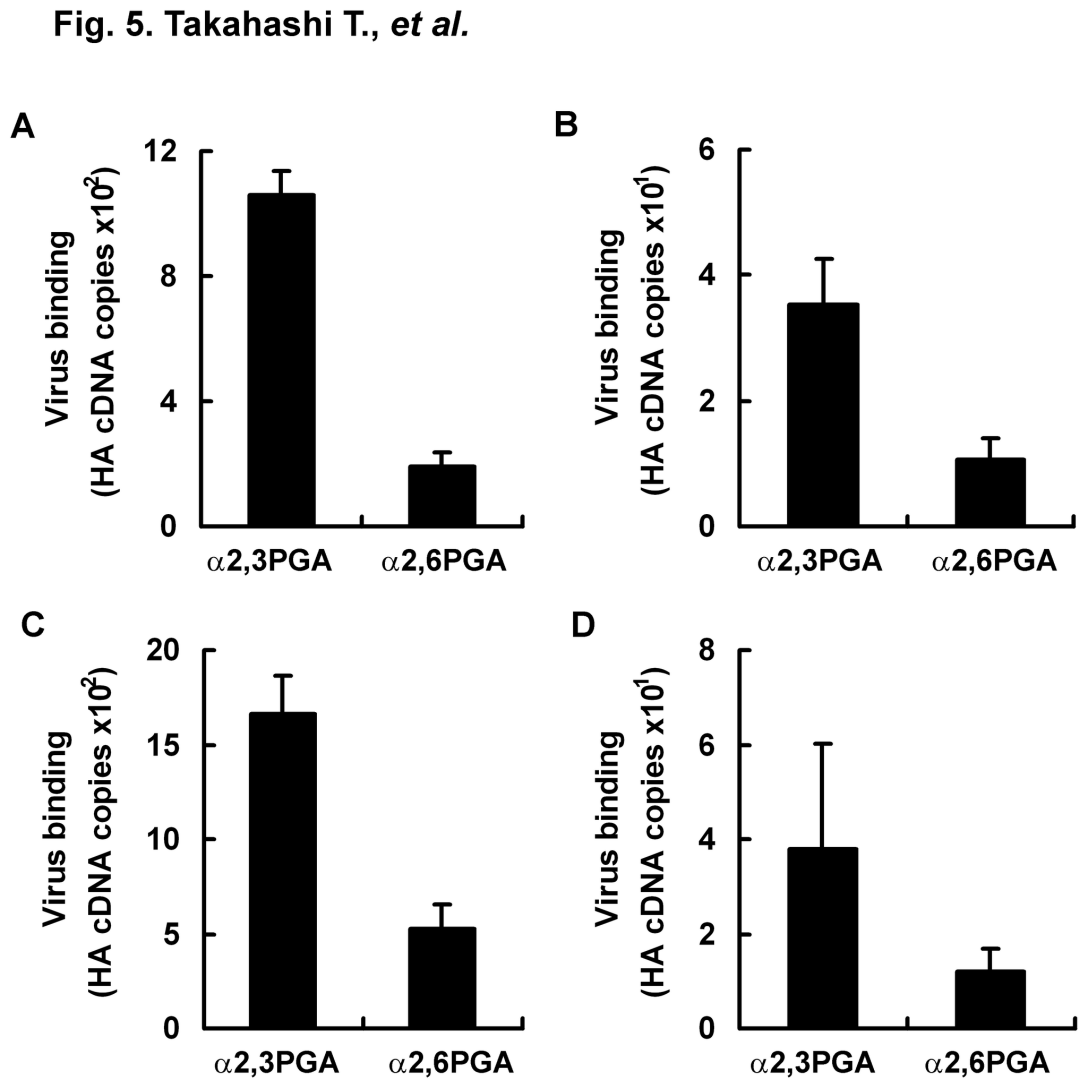
Direct detection of receptor binding specificity of HPAIs in swabs by real-time PCR. A microplate was immobilized with 200 ng/well of α2,3PGA or α2,6PGA and then blocked with 1% lipid-free BSA in PBS at 4 °C overnight. Trachea swabs (A and C) and cloaca swabs (B and D) were obtained from chickens infected with HPAIs CS10 (A and B) and CM11 (C and D). Swabs were suspended in equal amounts of PBS. The swab suspensions (50 μl/well) were incubated for 2 h at 4 °C on microplates that were immobilized with α2,3PGA or α2,6PGA. Viral RNAs of viruses bound to α2,3PGA or α2,6PGA were extracted. Viruses that bound to α2,3PGA or α2,6PGA were measured as copy number of HA cDNA by using real-time PCR. Each value (S.E.) is the average from three independent experiments. Directly from swabs obtained from HPAI-infected chickens, this assay indicated that HPAIs CS10 and CM11 had the general property of receptor binding specificity of avian IAV (preferential binding to Neu5Acα2,3Gal).

 We then tried to directly detect receptor binding specificity of HPAIs in clinical samples from chickens. For clinical samples, we used trachea swabs and cloaca swabs from chickens infected with the HPAI strain CM10 or CS11 reported previously [20]. Wells with immobilized α2,3PGA or α2,6PGA were blocked with lipid-free BSA and incubated with the supernatants of swabs after spin-down. HA cDNA copies of CM10 and CS11 were measured by using real-time PCR. Melting temperatures of all PCR products were confirmed to be similar to approximately 82 °C in a positive control (standard curve) of CM10 and CS11. HA cDNA copies of CM10 were 1.06 ×10^3^ (S.E. ± 7.87 ×10^1^) for α2,3PGA and 1.89 ×10^2^ (S.E. ± 4.93 ×10^1^) for α2,6PGA in trachea swabs, and they were 3.52 ×10^1^ (S.E. ± 7.22) for α2,3PGA and 1.05 ×10^1^ (S.E. ± 3.58) for α2,6PGA in cloaca swabs. HA cDNA copies of CS11 were 1.67 ×10^3^ (S.E. ± 1.98 ×10^2^) for α2,3PGA and 5.28 ×10^2^ (S.E. ± 1.23 ×10^2^) for α2,6PGA in trachea swabs, and they were 3.79 ×10^1^ (S.E. ± 2.23 ×10^1^) for α2,3PGA and 1.21 ×10^1^ (S.E. ± 4.59) for α2,6PGA in cloaca swabs. EID_50_ values in the cloaca swabs were approximately 10^1^- to 10^2^-times smaller than those in trachea swabs. This probably resulted in lower HA cDNA copies in cloaca swabs than in trachea swabs. Both CM10 in a trachea swab (Figure 5A) and a cloaca swab (Figure 5B) and CS11 in a trachea swab (Figure 5C) and a cloaca swab (Figure 5D) showed preferential binding to α2,3PGA compared to α2,6PGA, similar to the nature of almost all avian IAVs. This assay had adequate sensitivity for direct detection of receptor binding specificity of avian IAVs in clinical samples. 

The forward primer used in this one-step real-time RT-PCR against H5HA cDNA is conserved among many H5HA genes isolated from various hosts, including chickens, white peafowls, mynas, pigeons, crows, ducks, geese, tigers, leopards, and humans, in 1976, 1978, 2004, 2010, and 2011. The reverse primer against HPAI H5HA is conserved among many HPAI H5HA cDNAs from avian hosts, including chickens, ducks, swans, and owls, in 2007 to 2012 (in 100 hits on a search in NCBI nucleotide blast, http://blast.ncbi.nlm.nih.gov/Blast.cgi). These primers will be useful for detection of recent HPAI H5HA genes from wild birds and chickens since 2007.

## Discussion

 Traditional assays require high titers of influenza viruses, 2^5^ to 2^10^ HAU/50 μl for a solid-phase virus binding assay [9-11,18,19], 2^8^ to 2^10^ HAU/10 ml for a TLC virus overlay assay [11-13,23], and 2^8^ to 2^9^ HAU/ml or 10 μg/ml (approximately 2^8^ HAU/50 μl of A/Puerto Rico/8/1934 H1N1 strain) for a glycan array [15,16]. To obtain a high titer of virus, clinical samples must first be inoculated to embryonated eggs or cultured cells such as Madin-Darby canine kidney (MDCK) cells to replicate and collect virus stock (chorioallantoic fluid of an egg) as seed virus. Furthermore, seed virus of most virus strains including all subtypes must usually be propagated in a large amount of embryonated eggs or cultured cells to obtain a high titer of concentrated virus required for an adequate number of the traditional assays. Anti-IAV antibodies are required for detection of virus bound to sialo-glycoconjugates. However, since envelope glycoproteins of IAV strains are various in antigenicity (subtypes: HA 1 to 17, NA 1 to 10), a reactive antibody against the IAV strain tested must also be prepared. If a virus in clinical samples shows extremely efficient replication in eggs or cells, a high titer of seed virus may be able to be applied to traditional assays. However, seed virus and adequate amount of concentrated virus are usually required for traditional assays. In this case, it is estimated that traditional assays usually take more than one week from collection of a clinical sample to completion of measurement including the virus replication process to obtain a high titer of virus, as shown in Figure S2.

The new assay combined with real-time RT-PCR for receptor binding specificity of IAV was very sensitive and can be performed in a short time compared to the traditional assays. The new assay can be performed even with a low titer of IAV. Furthermore, it can detect receptor binding specificity of avian IAV directly from clinical samples of chickens, without the need for seed virus and virus replication to obtain a high titer of the virus. The virus is bound to sialo-glycoconjugates, a process that takes 2h. Viral RNA is extracted from virus bound to glycoconjugates using an RNA extraction kit, a process that takes less than 1 h, and measured with real-time PCR equipment using a one-step RT-PCR kit, a process that takes less than 1 h. The new assay takes a very short time, only less than 4 h, as shown in Figure S2. Additionally, preparation of embryonated eggs and maintenance of cultured cells are not required, unlike in the traditional assays.

 When a new subtype emerges (by replacement of a viral segmented genome between IAVs) or large antigenic alterations (by amino acid substitutions) of IAV occur, preparation of a specific antibody takes more than one month to obtain a large amount of antigen (viral proteins) and to immunize animals. Since gene sequence analysis requires only a very small amount of virus, preparation of specific primers takes only a few days. The new assay can early start, compared to traditional assays.

 When IAV replicates in embryonated eggs, there is a risk of HA mutation changing receptor binding specificity. A leucine-to-glutamine substitution at amino acid position 226 in HA (H3 HA numbering of human H3N2 IAV) has been reported to occur during virus replication in embryonated eggs and to shift receptor binding specificity of IAV to Neu5Acα2,3Gal from Neu5Acα2,6Gal [14]. Virus in clinical samples is thought to be mixed viruses carrying various mutations. Some particular viruses show preferential replication by selection of receptor properties in embryonated eggs or MDCK cells. The carbohydrate chain on asparagine at amino acid position 131 in HA (H1 HA numbering of A/USSR/90/77 H1N1 strain) interferes with binding to Neu5Acα2,6Gal, but it has little or no effect on binding to Neu5Acα2,3Gal. Lack of a carbohydrate chain by an asparagine-to-aspartate substitution at the position results in binding to Neu5Acα2,6Gal. For replication of mixed viruses with asparagine or aspartate at the site, the virus with aspartate shows selective replication in MDCK cells expressing Neu5Acα2,6Gal. On the other hand, the virus with asparagine shows selective replication in embryonated eggs abundantly expressing Neu5Acα2,3Gal [24]. Therefore, the virus replication process for a traditional assay has the risk of HA mutations and selective virus replication accompanying a change in receptor binding specificity. In the new assay, there is no risk of HA mutations, which were caused by the virus replication process to obtain a high titer of the virus.

In the case of simultaneous infection with more than two different subtypes of IAVs, receptor binding specificity of each strain could be detected by using subtype-specific HA primers. This assay would be beneficial as the primary screening method for detecting change in receptor binding specificities. 

## Conclusions

 HPAI infection, which has a mortality rate of approximately 60% in humans, shows frequent cases of human infections, especially in south-eastern Asia. Shift in receptor binding specificity of avian IAV from Neu5Acα2,3Gal to Neu5Acα2,6Gal is thought to be one of the major factors for acquisition of avian IAV transmission ability among humans. Surveillance of the shift in receptor binding specificity of HPAI is thought to be very important for prediction and prevention of a catastrophic pandemic of HPAI among humans. The new assay enables direct detection of receptor binding specificity of HPAI in poultry clinical samples. The assay also has some valuable advantages compared to traditional assays, as summarized in Table 2. Notably, detection of receptor binding specificity of HPAI by the new assay can be completed in a very short time (only less than 4 h), unlike the traditional assays (more than one week). Our receptor binding assay combined with real-time RT-PCR would be a beneficial tool for primary screening in numerous clinical samples to investigate a shift in receptor binding specificity of HPAI from Neu5Acα2,3Gal to Neu5Acα2,6Gal.

**Table 2:** Valuable advantages of new assay compared to traditional assay.

Comparison point	Traditional assay	New assay
HA mutation caused by virus replication process		
	Risk	No risk
Time required	More than one week	Less than 4 h
Virus detection tool	Antibody	Primer
Virus replication	Required (maintenance of eggs or cells)	Not required
Virus titer	high	low

## Supporting Information

Figure S1 **Structure of PGAs.** PGAs have an LacNAc chain, the terminal of which binds with Neu5Ac by α2,3 linkage (A) or α2,6 linkage (B).
(TIF)

Figure S2 Comparison of new assay with traditional assay in a required time.
(TIF)
