# Supplementary figures and images for: Sensitive and Direct Detection of Receptor Binding Specificity of Highly Pathogenic Avian Influenza A Virus in Clinical Samples
Source: PLoS One. 2013 Oct 18;8(10):e78125. doi: 10.1371/journal.pone.0078125 (PMC3799784; doi:10.1371/journal.pone.0078125)

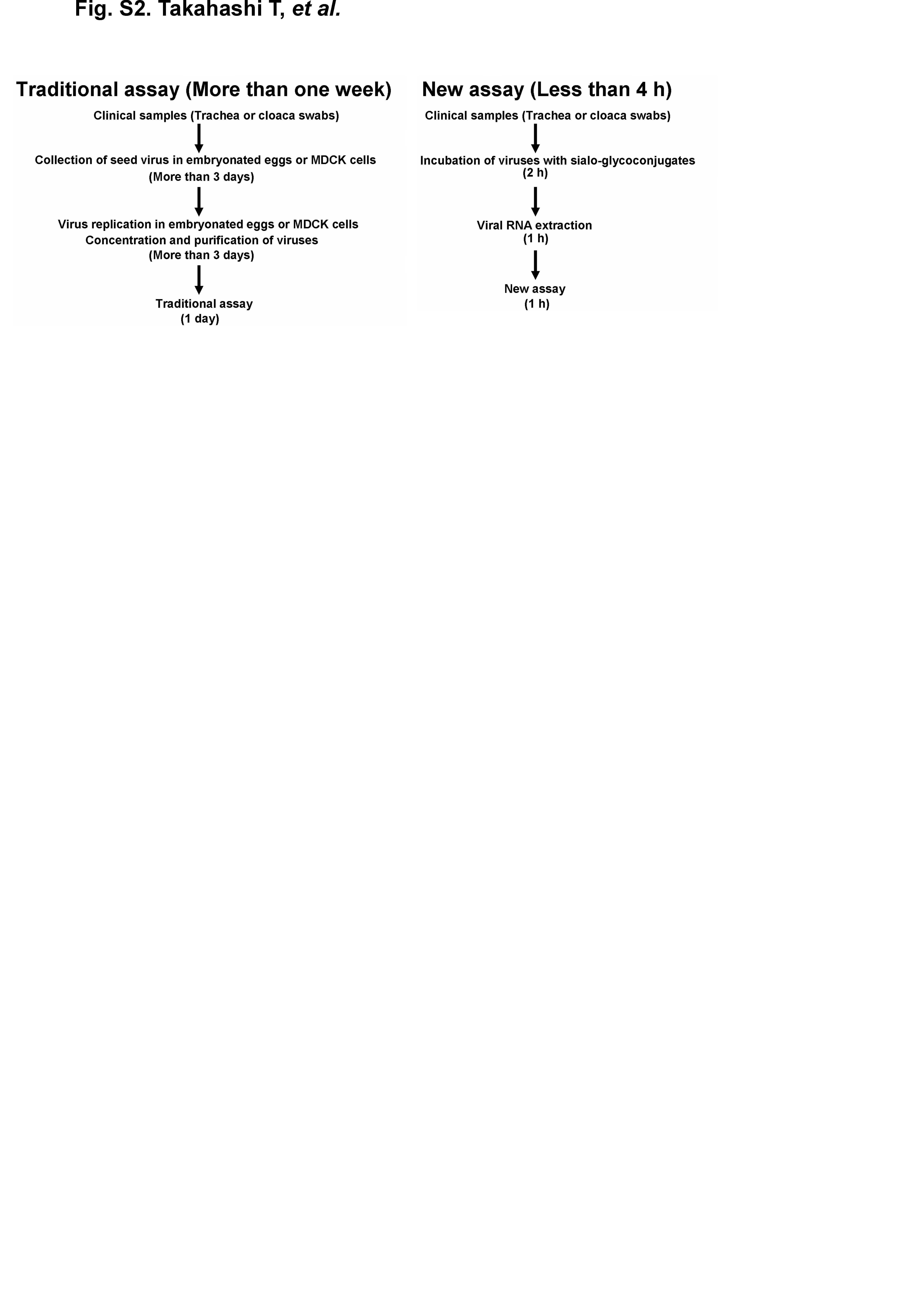

Supplement: Figure S2 — Comparison of new assay with traditional assay in a required time. (TIF) [file pone.0078125.s002.tif]
